# Efficacy and safety of microvascular decompression for hemifacial spasm: Endoscopy vs microscopy

**DOI:** 10.1097/MD.0000000000046920

**Published:** 2026-01-09

**Authors:** Moukun Liu, Jiangu Gong, Yuanliang Ye, Ruixiang Wei, Jinhuan Liu, Xuejing Wei

**Affiliations:** a Department of Neurosurgery, Engineering Technological Research Center for Nervous Anatomy and Related Clinical Applications, Liuzhou, Guangxi Autonomous Region, China; b Department of Human Anatomy, Guangxi Medical University, Nanning, Guangxi Zhuang Autonomous Region, China; c Department of Neurosurgery, Liuzhou People’s Hospital, Liuzhou, Guangxi Autonomous Region, China; d Department of Cadre Healthcare, Liuzhou People’s Hospital, Liuzhou, Guangxi Autonomous Region, China.

**Keywords:** complications, endoscope, hemifacial spasm, microvascular decompression

## Abstract

Microvascular decompression (MVD) is the most effective approach to treating hemifacial spasm (HFS). We aimed to evaluate the clinical efficacy and safety of endoscopic MVD (E-MVD) for treating HFS versus microscopic MVD (M-MVD). We analyzed consecutive 189 HFS individuals who underwent either an E-MVD or M-MVD procedure (January 2016–June 2024). The efficacy was assessed by operation time, learning curve, and clinical efficacy. Safety was assessed by the duration of dizziness and postoperative complications. We found none of the groups showed significantly different clinical efficacy (*P* = .709) after 1 year of follow-up. No statistical difference was presented in postoperative complications between both groups. The E-MVD group arrived at the plateau period of the learning curve at a quicker pace. The incidence of dizziness postoperatively in the E-MVD group is less than that in the M-MVD group (*P* = .037). E-MVD has the same clinical efficacy as M-MVD and reduces the learning curve. Further investigation needs to be conducted, preferably in a randomized controlled setting.

## 1. Introduction

Hemifacial spasm (HFS) mainly manifests as involuntary paroxysmal tonic or clonic movements on 1 side of the face. HFS results from arterial or venous vascular compression of the facial nerve (FN) root entry zone.^[1,2]^ Vessel compression is believed to result in nerve demyelination, which alters signal transmission, leading to muscle spasms in the territory that is innervated by the FN.^[3]^ Microvascular decompression (MVD) surgery can be safely and effectively used to treat HFS via the retrosigmoid suboccipital approach,^[4,5]^ which involves lifting the compressing vessel and inserting a Teflon material between the nerve and the compromising vessel.^[6]^ The clinical symptoms can be alleviated by isolating the offending blood vessels from the FN.^[7]^ Inadequate surgical anatomy visualization, especially at the position of contact between the offending vessel (OV) and the root entrance zone, can lead to treatment failures.^[8–11]^ This observation shows that there is an issue with the visualization under the microscope.^[12]^ More anatomical separation is required to visualize the desired anatomy, and direct vision of the light source was a prerequisite when using the microscope. In addition, it is a challenge for young doctors to comprehend the anatomy of the FN that enters/exits the brainstem, resulting in a lengthy learning curve.

Recently, endoscopic methods, including endoscopic or endoscope-assisted MVD, have been employed for MVD procedures.^[13–17]^ With the maturation of the technique and the development of endoscopic surgery skills, certain drawbacks of microscopic MVD (M-MVD) can be mitigated. Numerous publications have demonstrated the enhanced efficacy of endoscopic surgery for trigeminal neuralgia in identifying the problematic site of neurovascular conflict relative to microscopy.^[18,19]^

Herein, endoscopic surgery was applied for individuals with HFS, and evaluated its safety and effectiveness compared to microscopic procedures.

## 2. Materials and methods

### 2.1. Patients

**Figure 1.**
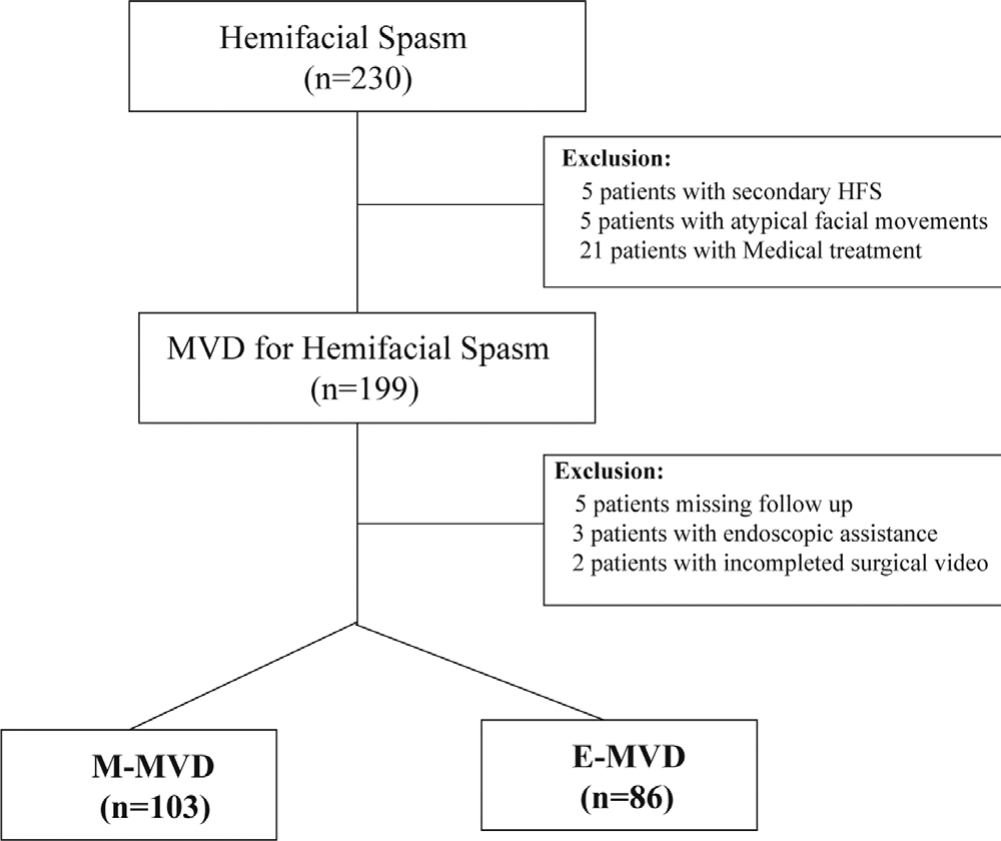
STROBE flow chart of patients with hemifacial spasm.

Between January 2020 and June 2024, 86 consecutive patients with HFS received the endoscopic procedure (E-MVD). The patients in the E-MVD group were matched with a cohort of consecutive 103 individuals who underwent M-MVD (January 2016–December 2019; Fig. 1). The demographic and clinical features of individuals, encompassing age, gender, lesion laterality, kind of OV, intraoperative observations, surgical results, and complications, were documented. The care unit, medical, and operating room protocols were unchanged during both research periods. Both patients with M-MVD and E-MVD was operated on by the same senior doctor (Dr Ye). The E-MVD cohort was matched with a M-MVD cohort patient who was similar in terms of baseline features, such as patient age, gender, OV, duration of symptoms, etc. The study was approved by the ethics committee of Liuzhou Hospital in agreement with the Declaration of Helsinki. All study was conducted in compliance with relevant instructions and rules. The ethics committees acquired informed consent. The patient or family members provided written consent before the surgery.

### 2.2. Inclusion and exclusion criteria

HFS diagnosis was conducted by the clinical histories and outcomes of individuals from physical tests conducted by 2 qualified clinicians. HFS severity was categorized according to Cohen’s criteria (grades 1–4): enhanced blinking induced by external stimuli; mild, detectable fluttering, not debilitating; moderate, highly obvious spasms, slightly debilitating; extremely debilitating (e.g., inability to drive or read). Grades 1 to 2 indicate moderate, while grades 2 to 3 indicate severe. Exclusion criteria: patients with secondary HFS and atypical facial movements; patients did not have a follow-up post-surgery; the family or legal representative of the patient did not consent to surgery.

### 2.3. Intraoperative neuromonitoring

We consistently observed abnormal muscle responses (AMR) and baseline electrocardiograms (BAEPs) during the surgical procedure. Nerve stimulation of the ipsilateral mentalis muscle and the marginal mandibular branch of the ipsilateral FN were used to record AMRs from the ipsilateral orbicularis oculi and mentalis muscles, respectively. Both left and right ears were recorded using channels A1-CPz and A2-CPz, with changing condensation and rarefaction clicks administered at a frequency of 17 Hz. With a sound pressure of 80 dB, the process was carried out without any interference from either side. The application of 40 dB white noise on the other ear served to obfuscate crossover reactions. All BAEPs of 50 to 2000 Hz were filtered using bandpass filtration, and 200 to 1000 trials were used to record them. We measured the longest latency and smallest amplitude of wave *V* in BAEPs by calculating the maximum alteration from the baseline waveform. The value of ([maximum change value−baseline value]/baseline value) was utilized to detect the latency and amplitude change rate. A significant BAEP alteration is characterized by an elevation in wave *V* latency of >1.0 ms and a reduction in amplitude of >50%.

### 2.4. Surgical procedures

**Figure 2.**
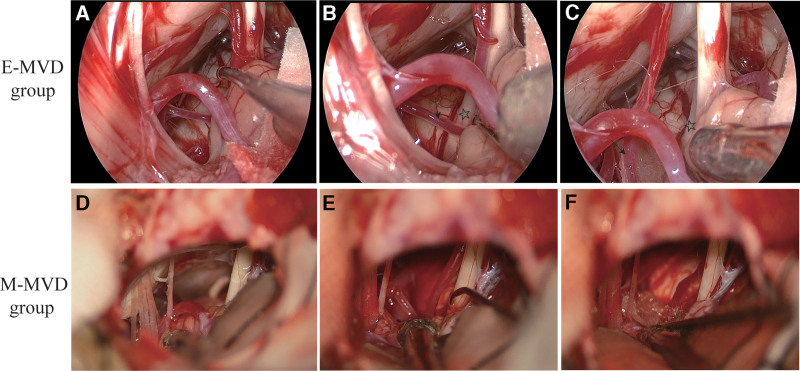
Endoscopic surgery for HFS. Expose the cerebellar glomerulus by separating the arachnoid membrane (A). The branch of the anterior inferior cerebellar artery (AICA) and the root exit zone of the FN (star) were displayed clearly (B). The AICA (arrow) was removed, and a small piece of Teflon felt was placed (C). Microscopic surgery for HFS. The auditory nerve and the posterior inferior cerebellar artery (PICA) were revealed by fully separating the arachnoid membrane (D). Due to the obstruction of the flocculus, the root exit zone of the FN cannot be displayed completely (E). The AICA branch is repositioned towards the skull base by interposing a thin piece of Teflon felt between the artery and the brainstem (F). AICA = anterior inferior cerebellar artery, FN = facial nerve, HFS = hemifacial spasm, PICA = posterior inferior cerebellar artery.

Patients were positioned laterally under general anesthesia, with their heads secured in a Mayfield head frame (Integra Life Sciences Co., Wilmington). An incision was made in the skin that was retroauricular straight, measuring 4 cm. Then, bone was removed from the posterior margin of the sigmoid sinus, measuring 2 cm. A C-shaped incision was made in the dura. Finally, the dural flap was retracted laterally. In the E-MVD cohort, the procedure was executed with the 1-surgeon, 2-hand approach. The endoscope was fixed by electric retainers from Longchuang Co., China. Under endoscopy, the subarachnoid gap was dissected between the cochlear and glossopharyngeal nerves, facilitating the gradual release of cerebrospinal fluid (CSF). Once the FN and OV were determined, Teflon was used to dissect and replace the OV away from the root exit zone of the FN (Fig. 2A–C). The surgical area was re-irrigated, and a conclusive underwater assessment was performed to determine the sufficiency of decompressing. Duraplasty was implemented using a galeaperiost flap. In the M-MVD group, the FN and OV were exposed during the dissection of the arachnoid membrane using a microscope, and Teflon was then inserted between the vessels and brainstem (Fig. 2D–F). Keep a detailed record of the operative hemorrhage and duration of the procedure using a microscope or neuroendoscopy.

### 2.5. Postoperative management

Right after surgery, and also 3 days later, a CT scan of the brain was conducted. After the procedure, a lumbar catheter drain was placed to monitor CSF clarity. In case of an infection, the vertebral canal would be instilled with Amikacin (20 mg, administered 1–2 times a day).

### 2.6. Clinical follow‑up

The operative data encompassed the duration of the procedure (utilizing a microscope or endoscope), the volume of operative hemorrhage, postoperative duration, classification and quantity of OV, AMR, and vascular compression, all recorded post-surgery in accordance with published literature. Efficacy was evaluated based on the subsequent criteria. Complete remission, total eradication of spasm after surgery. Partial alleviation, nearly total cessation of spasms, with sporadic spasms manifesting under particular conditions, including stress. No remission, resulting in neither enhancement nor deterioration. Delayed remission: facial spasm did not disappear immediately after surgery. Spasm symptoms gradually disappeared within a few days to 3 months following surgery. Complications after operation comprised aseptic and bacterial meningitis, facial numbness, immediate and delayed facial paralysis, tinnitus, deafness, vertigo, diplopia, posterior cranial nerve damage, delayed wound healing, intracranial bleeding, subcutaneous effusion, and cerebral infarction. Postoperative dizziness was evaluated using the visual analog scoring method within a month of surgery. Scores above 60 that impact quality of life were assessed as positive. Staff, by means of a standardized script, carried out follow-up interviews over the phone or during outpatient visits to collect the data.

### 2.7. Statistical analysis

SPSS 22 for Windows (SPSS Inc., Chicago) was employed for statistical analysis. Continuous variables were denoted as mean ± standard deviation or median with interquartile range, based on distributing the variables. Categorical variables were denoted as frequencies or percentages. Pearson’s Chi-square tests were employed to detect statistical differences in proportions. Continuous variables were assessed via an independent *t* test or a Mann–Whitney *U* test. A *P*-value below .05 was considered significant.

## 3. Results

### 3.1. Baseline characteristics

A total of 189 individuals participated in the investigation, with 116 males and 73 females. The average age at diagnosis was 52.13 ± 9.62 years in the E-MVD group and 49.68 ± 10.01 in the M-MVD group. There were 86 patients undergoing E-MVD and 103 patients with M-MVD. No significant variations between the 2 groups in gender (*P* = .715), age (*P* = .080), disease duration (*P* = .489), and site of onset (*P* = .862; Table 1). No patients had experienced a transition from endoscopic surgery to microscopic surgery.

**Table 1:** General data of the 189 patients with hemifacial spasm.

Variable	E-MVD n = 86	M-MVD n = 103	*P* value
Mean age, yr*	52.13 ± 9.62	49.68 ± 10.01	.080
Male, no (%)	54 (62.79)	62 (60.19)	.715
Side of HFS, no (%)			.862
Left	44 (51.16)	54 (52.42)	
Right	42 (47.50)	49 (47.57)	
Duration of symptoms (mo)*	46.27 ± 39.19	49.87 ± 32.28	.489
Degree of HFS			.984
Moderate	26 (30.23)	31 (30.09)	
Severe	60 (69.77)	72 (69.90)	
Other chronic diseases, no (%)			
Hypertension	15 (17.44)	20 (19.41)	.851
Heart disease	2 (2.32)	7 (6.79)	.185
Diabetes	5 (5.58)	6 (5.82)	>.999
None	64 (74.41)	70 (67.96)	

HFS = hemifacial spasm, E-MVD = endoscopic microvascular decompression, M-MVD = microscopic microvascular decompression.

* Values are expressed as the mean ± SD.

### 3.2. Evaluation of efficacy

**Figure 3.**
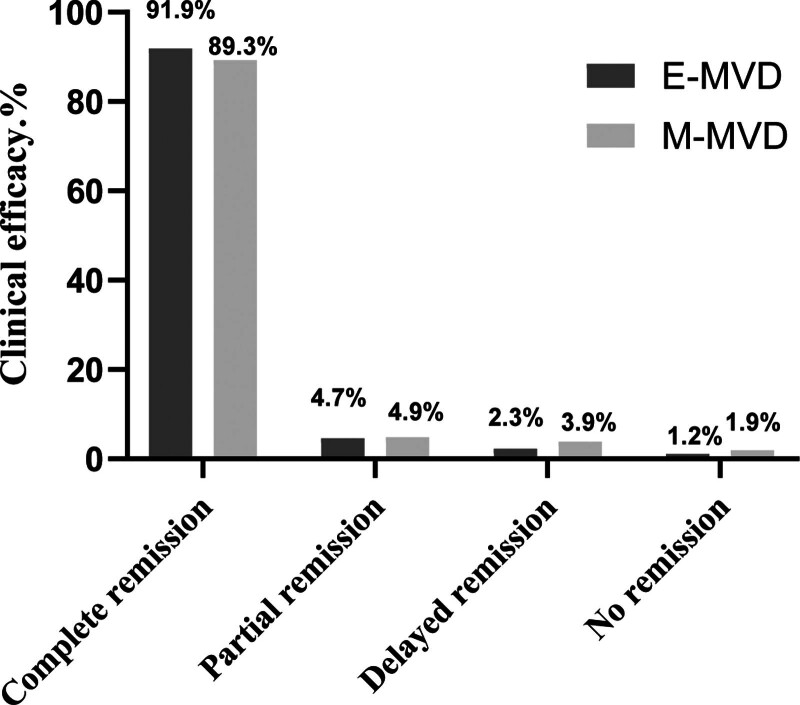
After 1 years of follow-up, the clinical efficacy was similar in both groups.

**Figure 4.**
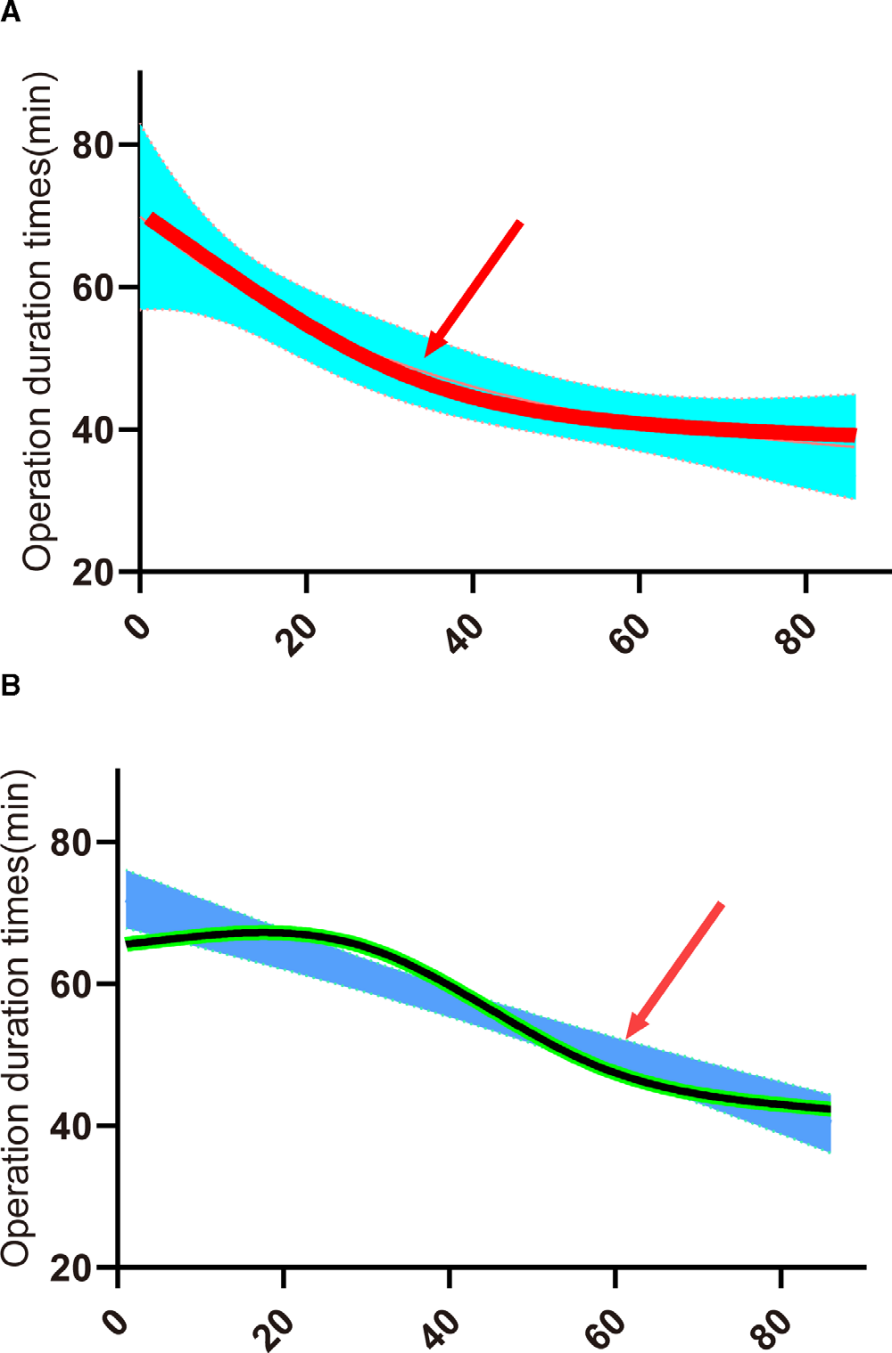
Compared with the M-MVD group (B), the E-MVD group (A) arrived at the plateau period of the learning curve at a quicker pace (Green arrow: turning point in the platform period). E-MVD = endoscopic microvascular decompression, M-MVD = microscopic microvascular decompression.

The intracranial operation duration times in the E-MVD group were (47.61 ± 17.48) minutes, which did not statistically differ from that of the times of (50.09 ± 13.35) minutes in the M-MVD group (*P* > .05). No significant variations were detected in the frequency of OV associated with the neurovascular conflict involving the FN between the 2 groups. The average relief rate of facial spasms in the E-MVD and M-MVD groups was 96.51% (95% CI: 0.99–0.90), and 94.17% (95% CI: 0.97–0.88), respectively (Fig. 3). After 1 year of follow-up, the E-MVD group had a similar recurrence rate compared with the M-MVD group [2.32% (95% CI: 8.08–0.41) vs 2.91% (95% CI: 8.22–0.79); Table 2)]. The learning curve slopes for E-MVD and M-MVD were –0.334 ± 0.06 and –0.373 ± 0.04, respectively, with no significant variations. The E-MVD group reached the plateau period of the learning curve at a quicker pace than Group B (Fig. 4).

**Table 2:** Comparison of effective and safe results between 2 groups in patients with HFS.

	E-MVD	M-MVD	*P* value
Operation duration times (min)*	47.61 ± 17.48	50.09 ± 13.35	.076
Operative bleeding (mL)*	21.2 ± 9.62	23.12 ± 10.15	.186
Length of stay (d)*	11.78 ± 2.31	12.13 ± 1.23	.278
Offending vessels (%)			.167
AICA	19 (22.09)	24 (23.30)	
PICA	49 (55.81)	58 (56.31)	
VA	4 (4.65)	5 (4.85)	
Multiple	14 (16.28)	16 (15.54)	
Clinical efficacy (%)			.253
Complete remission	79 (91.86)	92 (89.32)	.624
Partial remission	4 (4.65)	5 (4.85)	.948
No remission	1 (1.16)	2 (1.94)	.66
Delayed remission	2 (2.32)	4 (3.88)	.543
AMR (%)			.321
Skin incision	84 (97.67)	100 (97.08)	
Arachnoid membrane	80 (93.02)	94 (91.26)	
End of surgery	5 (5.81)	7 (6.79)	
Recurrence rate after 1 yr, %	2 (2.33)	3 (2.91)	.802
Complications (%)			
Tinnitus	2 (2.32)	3 (2.91)	.802
Hearing impairment	3 (3.49)	4 (3.88)	.886
Facial paralysis	2 (2.32)	3 (0.29)	.802
Posterior cranial nerve injury	2 (2.32)	4 (3.88)	.543
Aseptic meningitis	10 (11.63)	14 (13.59)	.686
Dizziness	4 (4.65)	14 (13.59)	**.037**
Wound infection	1 (1.16)	2 (1.94)	.670
Subcutaneous effusion	2 (2.32)	3 (0.29)	.802
Length of headache (d)*	4.21 ± 1.15	4.89 ± 1.91	.096

Bold type indicates statistical significance.

AMR = abnormal muscle responses, AICA = anterior inferior cerebellar artery, PICA = posterior inferior cerebellar artery, E-MVD = endoscopic microvascular decompression, M-MVD = microscopic microvascular decompression.

* Values are expressed as the mean ± SD.

### 3.3. Evaluation of safety

**Figure 5.**
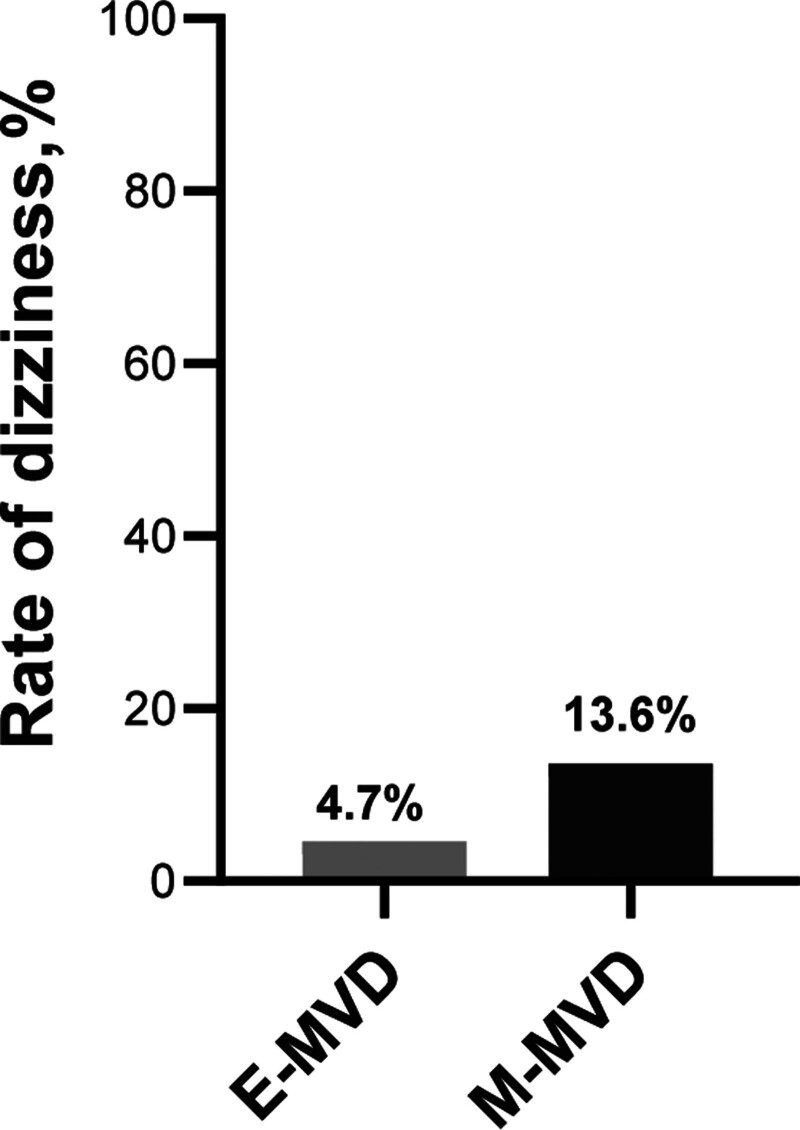
The dizziness postoperatively in the E-MVD group was less than that in the M-MVD group. E-MVD = endoscopic microvascular decompression, M-MVD = microscopic microvascular decompression.

No patients experienced diplopia, intracranial bleeding, or cerebral infarction in either group. The 2 groups exhibited no significant variations when comparing complications, including remission rate, AMR, length of postoperative headache, tinnitus, hearing impairment, facial paralysis, posterior cranial nerve injury, aseptic meningitis, subcutaneous effusion, and wound infection. Twenty-four patients with postoperative aseptic meningitis were cured by repeated lumbar punctures. Six patients with posterior cranial nerve injuries were treated with enteral nutrition therapy and rehabilitation therapy. All patients were cured after 3 weeks. There was a significant variation between both groups regarding postoperative dizziness (*P* = .037; 4.65% [95% CI: 0.114–0.018] vs 13.59% [95% CI: 0.215–0.083; Table 2; Fig. 5]).

## 4. Discussion

Herein, the efficacy and safety of E-MVD for HFS were examined by comparing it with M-MVD. We found that there was no difference in the clinical efficacy and complications comparing the E-MVD group with the M-MVD group. E-MVD reduces the learning curve and incidence of dizziness postoperatively.

Neuroendoscopic treatment for HFS has shown promising results, with a high rate of spasm resolution and minimal complications.^[20]^ Several studies have compared the efficacy and safety of neuroendoscopic surgery with traditional MVD. Neuroendoscopic surgery resulted in similar spasm resolution rates but with fewer complications, decreased operative time, and hospital stays compared to MVD.^[21,22]^ Some researchers reviewed the use of fully E-MVD in treating HFS using a meta-analyses. Fully E-MVD was found to be 93.3% effective in treating HFS, with most patients experiencing effective resolution at follow-up. Early postoperative complications were reported in 13.6% of patients, with transient facial palsy accounting for 6.8% and hearing impairment representing 4%. Fully E-MVD shows promising results in improving the quality of life for patients suffering from HFS.^[23]^ Some researchers were investigating whether M-MVD and E-MVD show better results than surgical treatments for hemifacial spasms in the Chinese population. They found that the effective rate for patients was 89% for M-MVD and 97% for E-MVD. The detection rate of offensive blood vessels was 91% for M-MVD and 98% for E-MVD. The recurrence rates were 5.7% for M-MVD versus 0.3% for E-MVD. The E-MVD group had a 5.1% incidence of facial paralysis or weakness and hearing loss, compared to the M-MVD group’s 12.1%. M-MVD and E-MVD had a similar incidence of cerebrospinal fluid leakage and intracranial infection.^[24]^

Endoscopic techniques aid in reaching optimal exposure in CPA surgery. The utilization of an endoscope offers a comprehensive view of the CPA cistern and facilitates significant flexibility in confined cisternal areas, hence enabling the surgeon to address most CPA lesions safely.^[25]^ The efficacy of the endoscope as an adjunct to the operating microscope in identifying the surgical anatomy of the cerebellopontine angle (CPA) was evaluated through examination of cadaveric specimens utilizing a microscope and 0° and 45° rigid endoscopes. The microscope offered adequate imaging of the posterior surfaces of the neuronal and vascular systems in the central region of the CPA cistern. In contrast, the endoscope offered superior visualization of the nerves’ junctions with the brainstem, their dural exits, and their vascular correlations. Additionally, the endoscope delivered enhanced views of the individual segments of the cerebellar arteries.^[26]^

The advantages of neuroendoscopic surgery over MVD can be attributed to several factors. First, the endoscope provides a broader perspective and better illumination, allowing for more precise identification and decompression of the OV. Second, the minimally invasive approach of neuroendoscopic surgery results in less tissue trauma and faster recovery times. Third, using an endoscope facilitates a better visualization of the CPA, which is crucial for the successful decompression of the FN.

The learning curve for MVD is influenced by several factors, including the surgeon’s familiarity with the anatomy of the CPA, the ability to recognize and decompress the OV, and the use of advanced surgical techniques and equipment. Several studies have investigated the learning curve of MVD for trigeminal neuralgia. The research adopted a retrospective analysis and collected data on MVD surgeries by a trainee and a senior neurosurgeon. Their findings indicated that the results and complication rates of the trainee were comparable to those of the senior. Also, shorter intervals between surgeries led to fewer complications and better outcomes.^[27]^ In our study, the endoscope’s ability to provide a clear view of the FN’s exit zone from the brainstem allows surgeons to more accurately identify the OV, thus contributing to a shorter learning curve for MVD surgery.

## 5. Limitation

The study’s limited sample size and lack of previous efficacy analysis could have an impact on its generalizability. A limited sample size may not be enough to detect clinically significant differences in outcome measures between the 2 groups. Single-center settings are potential for selection bias due to reasons such as surgeon preference or case complexity. A short follow-up may not be able to identify late recurrences or delayed complications. The retrospective design of the study includes possible biases, including recall bias and data omissions. Prospective, controlled trials would provide more robust evidence regarding the effectiveness and safety of E-MVD versus M-MVD. Future investigations with bigger, randomized cohorts are required to confirm the results.

## 6. Conclusion

E-MVD is safe and efficient in managing HFS, with a decreased learning curve and a reduction in postoperative dizziness. Our results require the support of multicenter, prospective, randomized, and controlled clinical trials.

## Author contributions

**Conceptualization:** Moukun Liu, Jiangu Gong, Yuanliang Ye, Xuejing Wei.

**Data curation:** Ruixiang Wei, Jinhuan Liu.

**Formal analysis:** Yuanliang Ye.

**Supervision:** Moukun Liu.

**Writing – original draft:** Xuejing Wei.

**Writing – review & editing:** Ruixiang Wei.

## References

[1] El Refaee E Marx S Rosenstengel C Baldauf J Schroeder HWS. Arachnoid bands and venous compression as rare causes of hemifacial spasm: analysis of etiology in 353 patients. Acta Neurochir (Wien). 2020;162:211–9. 31754846 10.1007/s00701-019-04119-5

[2] Cao C Li M Wu M Jiang X. Hemifacial spasm associated with the specific offending vein. Oper Neurosurg. 2025;28:337–46. 38995027 10.1227/ons.0000000000001284

[3] Iijima K Tajika Y Tanaka Y Yorifuji H Yoshimoto Y. Microanatomy around the facial nerve pathway for microvascular decompression surgery investigated with correlative light microscopy and block-face imaging. World Neurosurg. 2018;118:e526–33. 30257305 10.1016/j.wneu.2018.06.228

[4] Holste K Sahyouni R Teton Z Chan AY Englot DJ Rolston JD. Spasm freedom following microvascular decompression for hemifacial spasm: systematic review and meta-analysis. World Neurosurg. 2020;139:e383–90. 32305605 10.1016/j.wneu.2020.04.001 PMC7899163

[5] Lee HS Park K. Penetrating offenders in hemifacial spasm: surgical tactics and prognosis. Life (Basel). 2023;13:2021. 37895403 10.3390/life13102021 PMC10608199

[6] Grigoryan GY Dzhindzhikhadze RS Shumovsky VK Grigoryan YA. Interposition and transposition techniques of vascular decompression for hemifacial spasm. Interpozitsionnaya i transpozitsionnaya metodiki vaskulyarnoi dekompressii pri gemifatsial’nom spazme. Zh Vopr Neirokhir Im N N Burdenko. 2023;87:30–40. 37011326 10.17116/neiro20238702130

[7] Sindou M Mercier P. Microvascular decompression for hemifacial spasm: outcome on spasm and complications. A review. Neurochirurgie. 2018;64:106–16. 29454467 10.1016/j.neuchi.2018.01.001

[8] Li J Lyu L Chen C Yin S Jiang S Zhou P. The outcome of microvascular decompression for hemifacial spasm: a systematic review and meta-analysis. Neurosurg Rev. 2022;45:2201–10. 35048261 10.1007/s10143-022-01739-x

[9] Menna G Battistelli M Rapisarda A. Factors related to hemifacial spasm recurrence in patients undergoing microvascular decompression-a systematic review and meta-analysis. Brain Sci. 2022;12:583. 35624968 10.3390/brainsci12050583 PMC9139130

[10] Lee JM Park HR Choi YD. Delayed facial palsy after microvascular decompression for hemifacial spasm: friend or foe? J Neurosurg. 2018;129:299–307. 28862543 10.3171/2017.3.JNS162869

[11] Bigder MG Kaufmann AM. Failed microvascular decompression surgery for hemifacial spasm due to persistent neurovascular compression: an analysis of reoperations. J Neurosurg. 2016;124:90–5. 26295916 10.3171/2015.1.JNS142714

[12] Park CK Lee SH Park BJ. Surgical outcomes of revision microvascular decompression for persistent or recurrent hemifacial spasm after surgery: analysis of radiologic and intraoperative findings. World Neurosurg. 2019;131:e454–9. 31382068 10.1016/j.wneu.2019.07.191

[13] Zheng X Zhang B Shao D. Fully endoscopic microvascular decompression for hemifacial spasm: a clinical study and analysis. Neurosurg Rev. 2024;47:83. 38363437 10.1007/s10143-024-02311-5 PMC10873216

[14] Lehmann S Schroeder HWS. Endoscope-assisted microvascular decompression in hemifacial spasm: 2-dimensional operative video. Oper Neurosurg. 2023;25:e79. 37350594 10.1227/ons.0000000000000680

[15] Ricci G Di Stadio A D’Ascanio L. Endoscope-assisted retrosigmoid approach in hemifacial spasm: our experience. Braz J Otorhinolaryngol. 2019;85:465–72. 29784621 10.1016/j.bjorl.2018.03.015 PMC9443034

[16] Ajmera S Blue R Lee JYK. Endoscopic microvascular decompression. Adv Tech Stand Neurosurg. 2024;52:245–52. 39017798 10.1007/978-3-031-61925-0_17

[17] Peng W Zhao R Guan F. Fully endoscopic microvascular decompression for the treatment of hemifacial spasm, trigeminal neuralgia, and glossopharyngeal neuralgia: a retrospective study. BMC Surg. 2023;23:331. 37891595 10.1186/s12893-023-02214-0 PMC10612333

[18] Yu G Xia Y Gong W Min F Leng J Xiang H. Comparison of the efficacy of complete endoscopic and microscopic vascular decompression in the treatment of classical trigeminal neuralgia. World Neurosurg. 2024;190:e212–22. 39032638 10.1016/j.wneu.2024.07.094

[19] Li Y Mao F Cheng F Peng C Guo D Wang B. A meta-analysis of endoscopic microvascular decompression versus microscopic microvascular decompression for the treatment for cranial nerve syndrome caused by vascular compression. World Neurosurg. 2019;126:647–55.e7. 30776512 10.1016/j.wneu.2019.01.220

[20] Flanders TM Blue R Roberts S. Fully endoscopic microvascular decompression for hemifacial spasm. J Neurosurg. 2019;131:813–9. 30497190 10.3171/2018.4.JNS172631

[21] McGahan BG Albonette-Felicio T Kreatsoulas DC Magill ST Hardesty DA Prevedello DM. Simultaneous endoscopic and microscopic visualization in microvascular decompression for hemifacial spasm. Oper Neurosurg. 2021;21:540–8. 34662911 10.1093/ons/opab348

[22] Yang D Shu W Du T Li J Zhu H. Safety and efficacy of endoscope-assisted versus microscopic microvascular decompression surgery for hemifacial spasm: a prospective cohort study. Acta Neurol Belg. 2024;124:1555–60. 38625498 10.1007/s13760-024-02539-4

[23] Ansari A Tavanaei R Alikhani A. Fully endoscopic microvascular decompression for hemifacial spasm: a systematic review. Neurosurg Rev. 2025;48:285. 40050528 10.1007/s10143-025-03181-1

[24] Zhao Z Chai S Xiao D. Microscopic versus endoscopic microvascular decompression for the treatment of hemifacial spasm in China: a meta-analysis and systematic review. J Clin Neurosci. 2021;91:23–31. 34373033 10.1016/j.jocn.2021.06.034

[25] Xie Z Zhuang Y Liu J. Fully endoscopic neurosurgery using a two-handed technique for cerebellopontine angle tumors via the retrosigmoid approach. Front Oncol. 2024;14:1485932. 39737402 10.3389/fonc.2024.1485932 PMC11683133

[26] Takemura Y Inoue T Morishita T Rhoton AL Jr. Comparison of microscopic and endoscopic approaches to the cerebellopontine angle. World Neurosurg. 2014;82:427–41. 23891582 10.1016/j.wneu.2013.07.013

[27] Phang SY Martin J Zilani G. Assessing the safety and learning curve of a neurosurgical trainee in performing a microvascular decompression (MVD). Br J Neurosurg. 2019;33:486–9. 31111746 10.1080/02688697.2019.1617401

